# Supplementary material for: Flexible Wet-Spun PEDOT:PSS Microfibers Integrating Thermal-Sensing and Joule Heating Functions for Smart Textiles
Source: Polymers (Basel). 2023 Aug 17;15(16):3432. doi: 10.3390/polym15163432 (PMC10457801; doi:10.3390/polym15163432)
Supplement: Supplementary file 1 [file polymers-15-03432-s001.zip › polymers-2544264-supplementary.pdf]

Supplementary Materials for

Flexible Wet-Spun PEDOT:PSS Microfibers Integrating Thermal-Sensing and Joule Heating Functions for Smart Textiles

Yan Li [1], Hongwei Hu [2,*], Teddy Salim [3], Guanggui Cheng [2,*], Yeng Ming Lam [3] and Jianning Ding [2,4]

[1] School of Mechanical Engineering, Jiangsu University of Science and Technology, Zhenjiang, 212003, China; yanli@just.edu.cn
[2] Institute of Intelligent Flexible Mechatronics, School of Mechanical Engineering, Jiangsu University, Zhenjiang, 212013, China; hwhu@ujs.edu.cn, ggcheng@ujs.edu.cn
[3] School of Materials Science and Engineering, Nanyang Technological University, 50 Nanyang Avenue, 639798, Singapore; tsalim@ntu.edu.sg
[4] School of Mechanical Engineering, Yangzhou University, Yangzhou, 225009, China; dingjn@ujs.edu.cn
* Correspondence: hwhu@ujs.edu.cn (H. H); ggcheng@ujs.edu.cn (G. C)

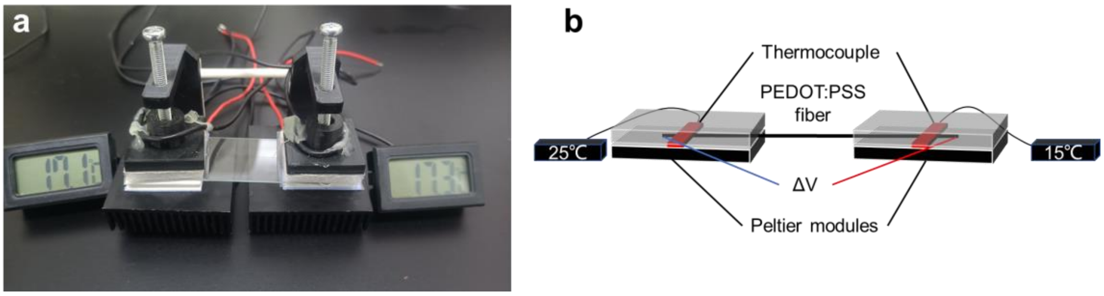

Figure S1. The homemade device used for measuring the Seebeck coefficiency of PEDOT:PSS fibers: the photo of the setup (**a**) and its schematic diagram (**b**).

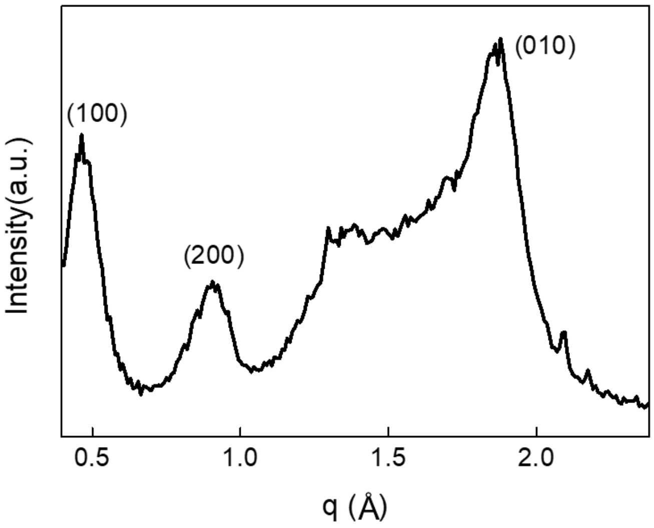

Figure S2. Plot of the integration of the scattering intensity in the horizontal direction in the WAXS image.
